# Broadband on-chip spectral sensing via directly integrated narrowband plasmonic filters for computational multispectral imaging

**DOI:** 10.1515/nanoph-2025-0398

**Published:** 2025-11-10

**Authors:** Qilin Zheng, Li Liang, Shunji Yang, Luyang Tong, Wenqiang Wang, Jibo Tang, Yu Zhang, Bintong Huang, Xiaobo He

**Affiliations:** Institute of Physics, 418471 Henan Academy of Sciences, Zhengzhou 450046, China; Zhejiang Engineering Research Center of MEMS, Shaoxing University, Shaoxing 312000, China; School of Physics and Optoelectronic Engineering, Foshan University, Foshan, Guangdong 528225, China

**Keywords:** sensing, spectroscopy, plasmonics filter, spectral imaging, on-chip

## Abstract

Spectroscopy underpins a wide range of applications, including biomedical diagnostics, precision agriculture, remote sensing, and industrial process control. Recent advances in silicon and microwave photonic integration have facilitated the miniaturization of spectroscopic systems, enabling portable, real-time analysis. However, the realization of a chip-scale platform that simultaneously achieves broadband coverage, high resolution, and scalable low-cost fabrication – particularly in the near-infrared (NIR) regime – remains a significant challenge. Here, we present a compact and cost-effective NIR spectroscopic sensing chip that monolithically integrates a plasmonic bandpass filter array with InGaAs photodetectors. The device is fabricated via single-step lithography and features a nanohole array with geometrically tunable narrowband transmission spanning 900–1,700 nm, exhibiting a full width at half maximum (FWHM) of 5.0 nm and a peak *Q*-factor of ∼284. The plasmonic filters are directly integrated with the detectors through a SiN_*x*_ spacer layer, eliminating post-fabrication alignment and enhancing scalability. A 16-channel super-pixel layout, combined with computational spectral reconstruction, enables ∼1 nm resolution near 1,550 nm and supports high-fidelity spectral imaging. This work demonstrates a scalable, detector-compatible approach to on-chip NIR spectroscopy, offering a promising route toward deployable, compact spectral sensing platforms.

## 1 Introduction

Spectroscopic sensing technologies offer high precision, low latency, imaging capability, and multidimensional detection, making them indispensable in critical fields such as remote sensing, geological exploration, petrochemical processing, industrial inspection, and biomedical diagnostics [1], [2], [3], [4]. Near-infrared (NIR) spectroscopy has garnered significant attention in biomedical applications due to its rich biochemical fingerprint features [5], [6], [7], [8]. Since the 1990s, NIR spectroscopy has evolved into a core analytical tool across agriculture, medicine, environmental monitoring, and food safety, valued for its non-destructive, rapid, and highly sensitive detection capabilities [9], [10], [11]. The advancement of micro/nanophotonics and information technology, coupled with the growing demand for compact, lightweight platforms – such as unmanned aerial vehicles and wearable devices – has accelerated the miniaturization and integration of optical sensors [12], [13]. However, current spectrometers are largely based on bulky and expensive benchtop systems, such as Fourier-transform infrared (FTIR) and grating-based NIR spectrometers, which are poorly suited for in-field or mobile applications. This limitation has hindered the widespread adoption of NIR spectroscopy in practical scenarios [1].

Recent developments in nanophotonic and microfabrication technologies have opened new avenues for chip-scale spectroscopic sensing. These structures enable precise subwavelength light manipulation and enhanced light–matter interaction, promising high sensitivity and resolution in a significantly reduced footprint [14]. Nevertheless, many miniaturized designs primarily focus on physical downscaling without fundamentally rethinking the optical architecture, often resulting in compromised performance. For instance, miniaturized diffraction gratings typically suffer from reduced spectral dispersion efficiency and signal-to-noise ratio, exposing a key challenge: achieving high resolution and sensitivity in space-constrained systems. Computational spectrometers-on-chip have emerged as a promising solution, harnessing algorithmic reconstruction to decouple system performance from physical constraints [15], [16], [17], [18], [19], [20], [21], [22], [23], [24], [25]. These devices leverage advanced materials and micro/nanostructures, along with machine learning and signal processing, to enable integrated and reconfigurable spectroscopic functionality. Research in this area broadly falls into two directions: advanced materials and advanced nano-optical structure.

In the realm of materials innovation, bandgap-tunable nanomaterials offer powerful routes to spectral selectivity. Techniques such as compositional tuning [26], [27], bias-induced modulation [28], [29], phase-change materials [30], and organic semiconductors [31] have enabled tunable spectral responses. For instance, Bao et al. developed a miniaturized spectrometer using 195 types of colloidal quantum dots (CQDs) integrated with CCDs, achieving tunable bandgaps over a 300 nm range [26]. Yang et al. synthesized CdS_*x*_Se_1−*x*_ nanowires with a spatially varying bandgap from 1.74 to 2.42 eV, enabling 10 nm resolution across 500–630 nm [27]. Sun et al. demonstrated bias-controlled perovskite photodetectors for spectral tuning via ion migration, achieving ∼5 nm resolution [27]. In the mid-infrared, Kim et al. employed Ge_2_Sb_2_Te_5_ (GST) phase-change materials within plasmonic nanohole arrays to realize broadband spectral tuning across 2.91–3.41 μm [30]. Other approaches include exploiting van der Waals heterostructures [28], [29], heavy-metal atom doping [32], and organic narrowband absorbers [31]. However, many of these materials face challenges in stability, scalability, or fabrication complexity.

In advanced nano-optical structure, innovative photonic designs have demonstrated high-performance on-chip spectral filtering. For example, Cao et al. used helical waveguides in multimode planar waveguides to enhance optical path length, achieving 10 pm resolution within a 20 nm band [33]. CMOS-compatible dielectric metasurfaces have also shown great potential. Altug et al. integrated multispectral resonant metasurfaces with CMOS platforms, enabling high-resolution biosensing without mechanical scanning [34], [35]. Disordered nanohole arrays etched on SOI substrates have yielded 0.75 nm resolution at 1,550 nm via light scattering in photonic crystals, although their coupling efficiency remains limited [36]. Yu et al. employed photonic crystal slab arrays to couple incident light into in-plane propagation modes, forming random spectral fingerprints for compressed sensing, achieving 1 nm resolution with improved efficiency [37], [38]. Despite these advances, high-precision nanostructures typically demand strict control over geometric parameters, such as nanopillar diameter and periodicity, which poses scalability challenges. Moreover, compared to the visible range, progress in integrated infrared spectroscopy has been slower due to the high cost of focal plane arrays and the complexity of integrating IR-compatible spectral filters on-chip [3], [7], [19], [39]. These factors limit practical deployment in cost-sensitive or mobile environments.

**Figure 1:**
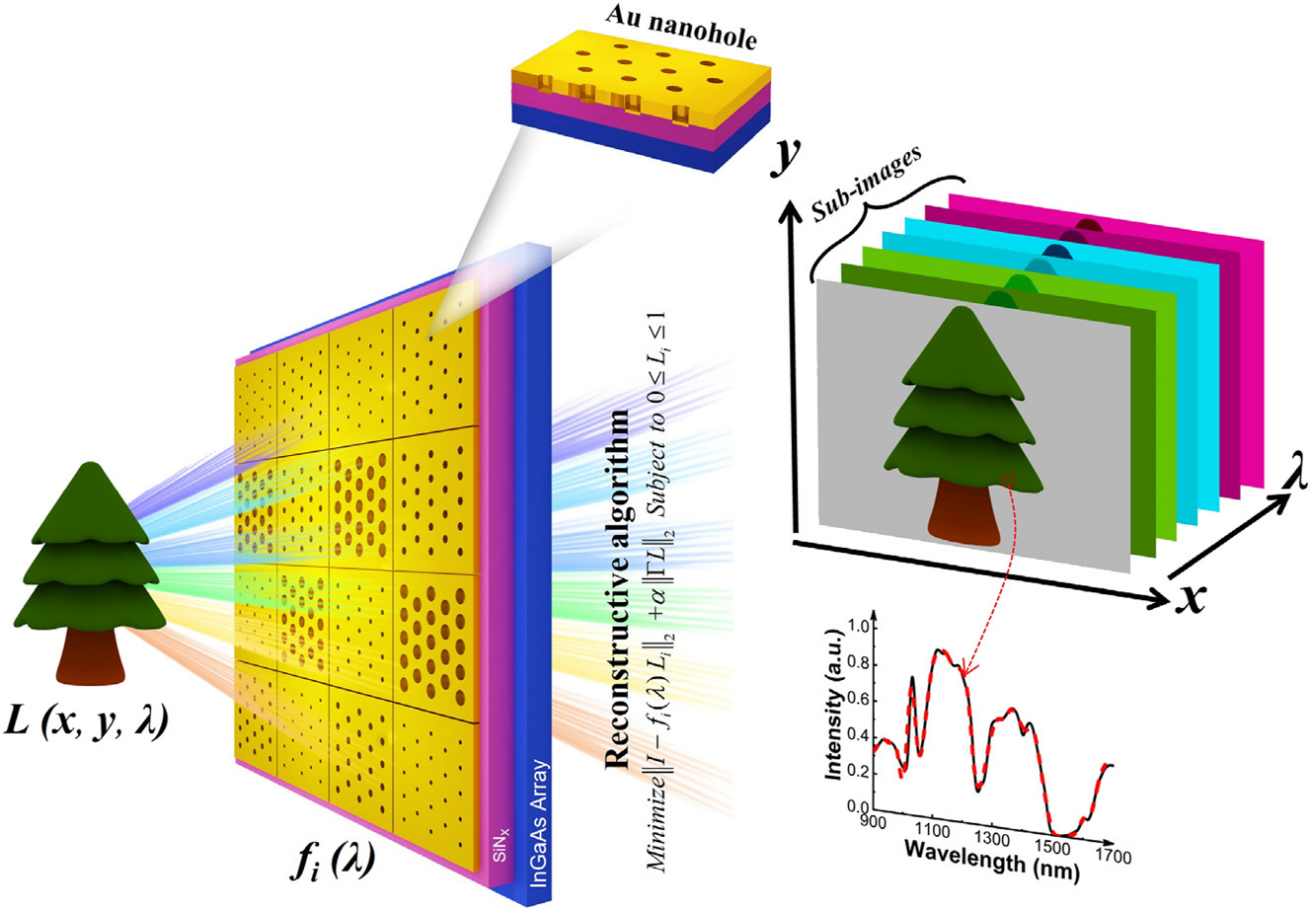
Conceptual diagram of the proposed broadband on-chip spectral sensing system. The spectral-spatial information of an object (*L*(*x*, *y*, *λ*), e.g., a tree scene) is converted to a 2D photocurrent map I (*x*, *y*) through a monolithically integrated chip *f*_*i*_(*λ*). This system features a vertically integrated architecture: Top: period-tunable plasmonic narrowband filter array implementing pixel-specific spectral encoding. Bottom: InGaAs photodetector array directly converting filtered light into electrical intensity signals.

With the rapid evolution of machine learning and computational optics, highly customized, low-channel-count spectrometers are becoming increasingly practical and economically viable. In this study, we report the development of a wafer-scale, low-noise plasmonic bandpass filter array operable from 900 to 1,700 nm. Fabricated via a single lithographic step, the array provides multiple passbands for integrated spectral sampling (Figure 1). We further direct integrate the filters with InGaAs photodetector arrays to form a compact 16-channel spectroscopic sensor with ∼1 nm resolution around 1,550 nm. Finally, we demonstrate computational spectral reconstruction and multispectral imaging, showcasing its potential as a scalable and multifunctional platform for near-infrared spectroscopic sensing.

## 2 Results

### 2.1 Narrowband near – infrared plasmonic bandpass transmission filter

**Figure 2:**
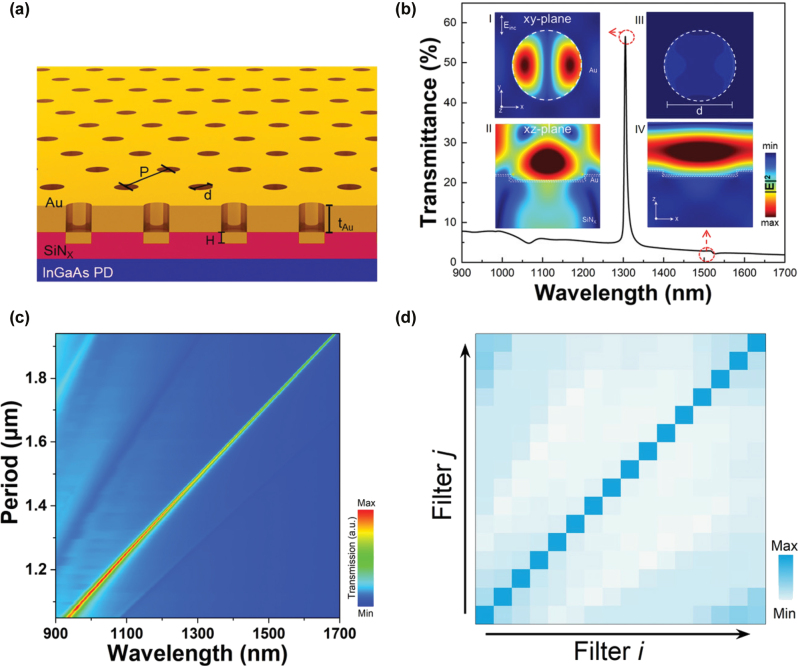
Period tunable narrowband near – infrared plasmonic bandpass transmission filters. (a) Schematic cross-section of the Au/SiN_*x*_ nanohole array structure. (b) Simulated transmission spectra for the device structure in (a). The nanohole period *P* = 1,500 nm, the depth *H* = 70 nm, the Au film thickness *t*_Au_ = 25 nm, and the fill factor *FF* = 0.7. Electric field distributions of device at characteristic wavelengths, I. *xy*-plane field intensity at 1,305 nm resonance wavelength (aperture center plane); II. *xz*-plane cross-section at 1,305 nm, illustrating the SPP resonance at the Au/SiN_*x*_ boundary within the nanohole array; III. *xy*-plane distribution at 1,500 nm off-resonance wavelength (>80 % intensity reduction); IV. *xz*-plane profile at 1,500 nm, showing the absence of boundary electric field enhancement under off-resonance conditions. (c) Calculated transmission spectra of filter arrays at *H* = 70 nm, *t*_Au_ = 30 nm and *FF* = 0.7. Chip is a 16-unit array with *P* = 1,050, 1,106, …, 1,950 nm. (d) The transmission spectra of the 16-period nanohole of the coefficient correlation matrix.

For on-chip spectroscopy, a high-efficiency narrowband bandpass filter operating across a broad wavelength range with low background has long been pursued as a pivotal component. We developed a novel plasmonic bandpass filter with simple architecture and direct detector integrability, comprising a dielectric nanohole array perforated in a thin metal film (Figure 2a). The resonant response arises from surface plasmon polariton excitation by the periodic nanohole array at the metal–dielectric interface [30], [40]. Shallow nanoholes (*H* < 80 nm) suppress out-of-plane radiative leakage, yielding a narrow transmission linewidth (FWHM < 10 nm). The optical response is primarily governed by three structural parameters: (i) Etching depth (*H*), which influences mode confinement and propagation loss; (ii) Au film thickness (*t*_Au_), which affects coupling efficiency and Ohmic dissipation; and (iii) Filling factor (*FF* = *d/P*, where *d* is the nanohole diameter and *P* is the period), which regulates inter-hole coupling and lattice diffraction. As quantitatively shown in Figures S1–3, these parameters collectively modulate the balance between resonant enhancement and intrinsic losses in the Au/SiN_*x*_ heterostructure, enabling wavelength-specific narrowband filtering across 900–1,700 nm. As shown in Figure 2b, a transmittance exceeding 55 % can be maintained while achieving an FWHM as narrow as 5 nm. Furthermore, the central wavelength of the bandpass filter can be precisely tuned by varying the nanohole periodicity, enabling wafer-scale fabrication of filter arrays through a single lithographic step.

**Figure 3:**
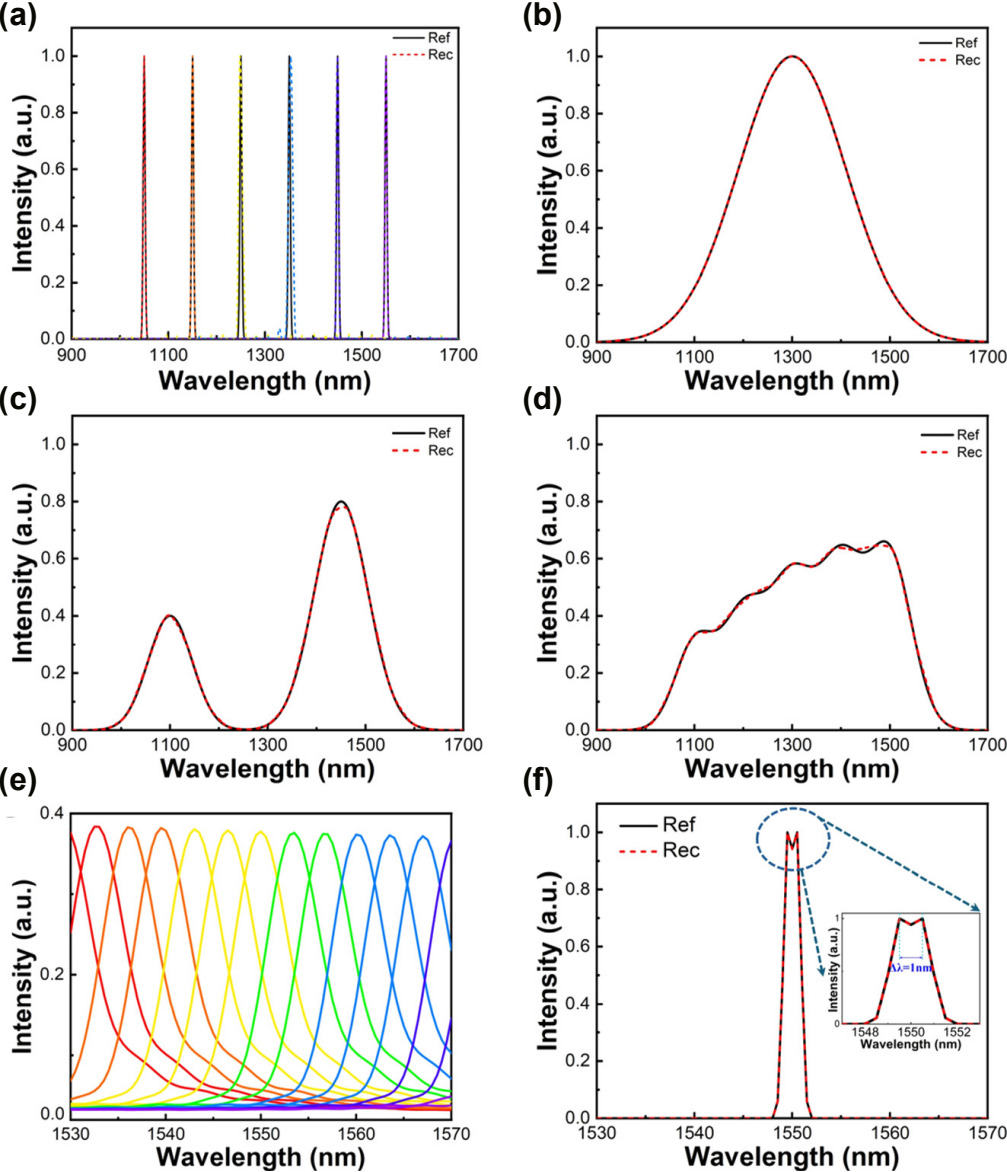
Spectral reconstruction comparisons to the reference spectrum. (a) Reconstructed target narrowband spectra (dotted lines, FWHM = 5 nm) using calculated filter responses as shown in Figure 3c. The black lines are the reference spectra. (b) Reconstructed single Gaussian peak target spectra (dotted lines, FWHM = 260 nm); (c) reconstructed two different half-width combinations target spectra (dotted lines, FWHM = 107/132 nm); (d) reconstructed target broadband spectra. (e) Transmission spectra generated by sixteen filter units with lattice periods ranging from 1.76 to 1.81 µm, covering the spectral range of 1,520–1,560 nm. (f) Reconstruction of a dual-peak signal centered at 1,550 nm with a 1 nm wavelength separation. The dashed box highlights the magnified view of the dual-peak region.

Building upon the optimized geometric parameters (*H* = 70 nm, *t*_Au_ = 25 nm, *FF* = 0.7, *P* = 1,500 nm), we numerically validated the plasmonic resonance filter via full-field electromagnetic simulations. Figure 2b demonstrates exceptional narrowband filtering across a 900–1,700 nm operational window: >55 % peak transmittance at resonance Ultra-narrow bandwidth (FWHM ≈ 5 nm, *Q* ≈ 130) Strong out-of-band rejection (<10 % transmittance at off-resonance wavelengths) Field confinement analysis reveals the physical origin: at resonance (1,310 nm), surface plasmon polaritons (SPPs) couple to Au-dielectric interfaces within the nanoholes, generating intense near-field enhancement (|*E*|/|*E*_0_| > 5.8). Conversely, off-resonance fields (1,500 nm) exhibit suppressed transmission (<10 %), confirming effective bandpass functionality. To extend spectral coverage, we engineered nanohole arrays with linearly scaled periods (*P* = 1.05–1.95 μm), achieving 16 discrete channels that quasi-continuously cover 900–1,700 nm (Figure 2c). This design enables computational spectrometry by constructing a high-dimensional spectral transfer matrix. Crucially, we evaluate the matrix quality through cross-correlation [2] analysis:

(1) $$C_{ij}=\frac{\int f_{i}\left(\lambda \right)f_{j}\left(\lambda \right)\text{d}\lambda }{\sqrt{\int f_{i}{\left(\lambda \right)}^{2}d\lambda \int f_{j}{\left(\lambda \right)}^{2}d\lambda }},$$

where *f*_*i*_(*λ*) denotes the transmission spectrum of the *i*-th (or *j*-th) filter, and *C*_*ij*_ quantifies the spectral orthogonality between channels, which forms the basis for high-accuracy spectral reconstruction. As quantified by the correlation matrix in Figure 3d, the filters exhibit ultra-low mutual correlation with a mean cross-correlation coefficient of 0.25.

Infrared spectroscopy provides critical molecular fingerprint identification for portable precision matter analysis. While rich in chemical information, conventional infrared spectrometers suffer from bulkiness, complexity, and high cost. Emerging Computational Spectroscopy chip-scale alternatives offer compelling advantages: ultracompact footprint, simplified optics, and high accuracy. Crucially, spectrometer performance hinges on two parameters: operational bandwidth and spectral resolution, both fundamentally governed by the filtering subsystem. Here, we introduce a plasmonic nanohole array serving as an integrated tunable filter that simultaneously delivers: Narrow linewidth (FWHM ≈ 5 nm, *Q*_max_ ≈ 284), broadband coverage (900–1,700 nm), high out-of-band rejection (>90 % suppression), enabling miniaturized computational spectrometers (Figure 2c).

Conventional grating spectrometers rely on spatially separated dispersive elements and long optical paths, with their resolution fundamentally constrained by detector pixel density. In contrast, our design integrates a periodically tunable transmission filter directly with the photodetector, thereby overcoming this limitation. This integration significantly reduces system complexity and footprint by eliminating bulky optical components. When paired with computational reconstruction, the architecture overcomes conventional resolution limits imposed by detector geometry.

**Spectral reconstruction methodology:**

For *N* = 16 filter channels spanning 900–1,700 nm (Figure 2c), the detector response is modeled as:

(2) $$I_{i}\;=\;\overset{\lambda \max}{\underset{\lambda \min}{\int }}L\left(\lambda \right)f_{i}\left(\lambda \right)\text{d}\lambda ,\;\;\;\;\left(i=1,2,\ldots ,16\right)$$

Here, *L*(*λ*) represents the target spectrum within the 900–1,700 nm range, while *f*_*i*_(*λ*) denotes the transmission response of the *i*-th plasmonic filter. The output signal from each detector corresponds to the convolution of *L*(*λ*) with its respective filter response. To construct the full sensing matrix, we precisely characterized *f*_*i*_(*λ*) using a wavelength-swept supercontinuum laser, scanning from 900 to 1,700 nm in 1-nm increments and recording each filter’s photoresponse. Recovering *L*(*λ*) from this under sampled dataset constitutes an ill-posed inverse problem, requiring advanced reconstruction techniques. To mitigate the influence of noise measurement, we apply *L*_2_-norm Tikhonov regularization for robust spectral reconstruction from undersampled data [22], [41]. The modified Tikhonov formulation employed in our work is given by:

(3) $$\text{Minimize}{\left\|I-fL\right\|}_{2}\;+\;\alpha {\left\|\Gamma L\right\|}_{2}\;\;\;\text{Subject}\;\;\text{to}\;\;0\leq L\leq 1,$$

where *α* is the regularization weight value, and Γ is the auxiliary matrix. As demonstrated in Figure 3a–d, the recovered spectra closely match the reference in both peak location and general profile, with only minor deviations. To quantitatively assess reconstruction accuracy, we use spectral fidelity, defined as:

(4) $$\text{Fidelity}=\frac{\overset{N}{\underset{i=1}{\sum }}\left(x_{i}\cdot {\tilde{x}}_{i}\right)}{\sqrt{\overset{N}{\underset{i=1}{\sum }}{x_{i}}^{2}\overset{N}{\underset{i=1}{\sum }}{{\tilde{x}}_{i}}^{2}}},$$

where *x*_*i*_ and ${\hat{x}}_{i}$ are the original and reconstructed spectral values at the *i*-th wavelength point, respectively, and *N* is the total number of data points. Fidelity values range from 0 to 1, with values approaching 1 indicating high reconstruction accuracy. Across multiple test spectra, our platform consistently achieves fidelity values exceeding 0.99, underscoring the high precision of the proposed spectroscopic architecture.

To evaluate the spectral resolution of our approach, we reconstructed a synthetic spectrum containing two closely spaced narrowband peaks (Δ*λ* = 1 nm) using the single-resonance response of our plasmonic filter structure, as shown in Figure 3f. Sixteen filter units, with periods ranging from 1.76 to 1.81 µm, were employed to generate transmission spectra spanning 1,520–1,560 nm (Figure 3e). Applying our reconstruction framework, we successfully resolved the characteristic double-peak profile using only 16 measurements, demonstrating the system’s high resolution. These results highlight the strong spectral discrimination capability of our plasmonic filter–InGaAs detector integrated chip, underscoring its potential for compact, on-site spectroscopic applications such as on-site material rapid inspection and point-of-care diagnostics.

**Figure 4:**
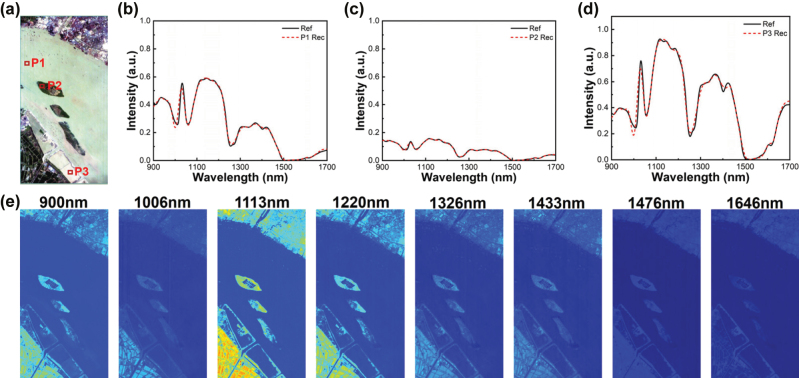
Hyperspectral imaging using the narrowband near-infrared plasmonic bandpass transmission filter spectrometer. (a) Hyperspectral data set of river image on May 3rd, 2013 [42]. (b–d) Recovered reflectance spectra (dotted lines) of three selected patches in the test multispectral image Figure 4a, the black lines are the reference spectra; (e) a series of reconstructed images at selected wavelengths. The intensity range of these images is normalized.

Building on the high-fidelity spectral reconstruction framework used in Figure 3, we further extend the plasmonic filter–detector integrated chip toward on-chip multispectral imaging, enabling spatially resolved spectral acquisition within a compact platform. This architecture uniquely combines: Monolithic integration of tunable nanohole filters with InGaAs photodiodes, computational multiplexing enabled by the 16-channel filters, snapshot spatial-spectral capture without mechanical scanning. Hyperspectral dataset validation: As shown in Figure 4a, a hyperspectral image consisting of 463 × 241 pixels across 76 spectral bands (900–1,700 nm) was reconstructed based on the HYPERION dataset acquired over Jiangsu Province, China, on May 3, 2013 [42]. This dataset, primarily intended for monitoring fluvial material variations, demands highly accurate and faithful spectral image reconstruction. Using the 16 filter channels described in Figure 2c, we performed pixel-wise spectral reconstruction across the full dataset. Figure 4b–d present representative spectra at three distinct locations, where the reconstructed results closely match the reference spectra, achieving a spectral fidelity greater than 0.99. In addition, spectral images at eight representative wavelengths were visualized, demonstrating excellent boundary fidelity of the river morphology and clearly delineated shoreline features (Figure 4e). Furthermore, silicon-based hot-carrier photodetectors offer a promising alternative to InGaAs detectors in the near-infrared range, substantially reducing cost and improving compatibility with standard semiconductor fabrication processes. In addition, the filter design exhibits strong spectral scalability: by tuning the structural period *P*, the operational band can be readily reconfigured to target specific wavelength ranges. Together, these results establish our chip-based platform as a compact and scalable solution for next-generation airborne or field-deployable imaging spectrometers.

## 3 Conclusions

In summary, we present a monolithically integrated narrowband plasmonic filter–detector chip that enables broadband computational spectroscopy across the near-infrared (900–1,700 nm) with high spectral fidelity. The device leverages a single-step lithographic fabrication of nanohole arrays, yielding geometrically tunable narrowband transmission with a FWHM of 5.0 nm and a *Q*_max_ of ∼284. Direct integration of Au nanostructures with InGaAs photodetectors via a SiN_*x*_ interlayer eliminates post-fabrication alignment, enhancing fabrication scalability and device robustness. Furthermore, a nonlinear parametric design framework effectively suppresses radiative and Ohmic losses, ensuring high transmission efficiency. The resulting 16-channel super-pixel architecture enables simultaneous point spectroscopy and snapshot hyperspectral imaging, demonstrating the potential of this compact platform for field-deployable and on-chip spectroscopic sensing applications. Future extensions may exploit higher *Q-*factor photonic strategies, such as bound states in the continuum (BIC), and metasurfaces to enhance spectral resolution, together with scalable design optimization to realize more spectral channels per super-pixel without compromising spatial resolution.

## Supplementary Material

Supplementary Material Details
