# Supplementary material for: Broadband on-chip spectral sensing via directly integrated narrowband plasmonic filters for computational multispectral imaging
Source: Nanophotonics. 2025 Nov 10;14(27):4957–64. doi: 10.1515/nanoph-2025-0398 (PMC12717906; doi:10.1515/nanoph-2025-0398)
Supplement: Supplementary file 1 — Supplementary Material Details [file j_nanoph-2025-0398_suppl_001.docx]

# **Broadband On-Chip Spectral Sensing via Directly Integrated Narrowband Plasmonic Filters for Computational Multispectral Imaging: Supporting information**

Qilin Zheng^1^*; Li Liang^2^; Shunji Yang^1^; Luyang Tong^1^; Wenqiang Wang^1^; Jibo Tang^1^; Yu Zhang^3^; Bintong Huang^1^; Xiaobo He^1^*

^1^Institute of Physics, Henan Academy of Sciences, Zhengzhou 450046, China

^2^Zhejiang Engineering Research Center of MEMS, Shaoxing University, Shaoxing 312000, China

^3^School of Physics and Optoelectronic Engineering, Foshan University, Foshan, Guangdong 528225, China

[*zhengqilin@hnas.ac.cn](mailto:*zhengqilin@hnas.ac.cn) [*hexiaobo@hnas.ac.cn](mailto:*hexiaobo@hnas.ac.cn)

**S1. Etching depth (*H*) dependence analysis**

The optical response of this narrowband plasmonic bandpass filter—consisting of a dielectric nanohole array perforated in a thin metal film with direct detector integrability—is primarily governed by three structural parameters: (i) etch depth *H*, which tunes out-of-plane radiation leakage and the coupling strength to surface plasmon polaritons (SPPs) at the continuous metal–dielectric boundary; (ii) Au film thickness *t_Au_*, which affects in-/out-coupling efficiency and Ohmic dissipation; and (iii) fill factor FF = *d/P* (with *d* the nanohole diameter and *P* the period), which regulates inter-hole coupling and lattice diffraction.

Figure S1 shows the simulated transmission evolution versus HHH. As HHH increases from 20 → 80 nm, the on-resonance peak near λ≈1305 nm rises from <15% to >55%, while the FWHM narrows from ~10 nm to ~4 nm. This improvement is attributed to (a) suppressed out-of-plane radiation leakage from the aperture region, and (b) enhanced overlap between the incident field and the SPP-mediated resonance at the Au/SiN_x_ boundary—together yielding stronger boundary field enhancement and a higher *Q-*factor. When *H* is further increased to 100–120 nm, the trend reverses: the transmittance drops to <40% and the FWHM broadens beyond 12 nm. Correspondingly, the filter *Q*-factor exhibits a non-monotonic dependence on HHH: increasing *H* from 10 → 70 nm elevates *Q-*factor from ~109 to a maximum of ~246 (Δ≈125%), whereas further etching to 120 nm reduces *Q-*factor to ~132. The degradation at large *H* arises mainly from: (i) increased Ohmic loss due to longer SPP energy flow paths and stronger metal overlap, and (ii) spectral broadening from additional leaky/parasitic channels and mismatch in the grating-assisted coupling condition.

Overall, these results identify an optimal etch-depth window of 60–80 nm, which balances boundary field enhancement against dissipative and radiative losses, and thus provides a practical guideline for realizing high *Q-*factor, SPP-mediated narrowband plasmonic filters.


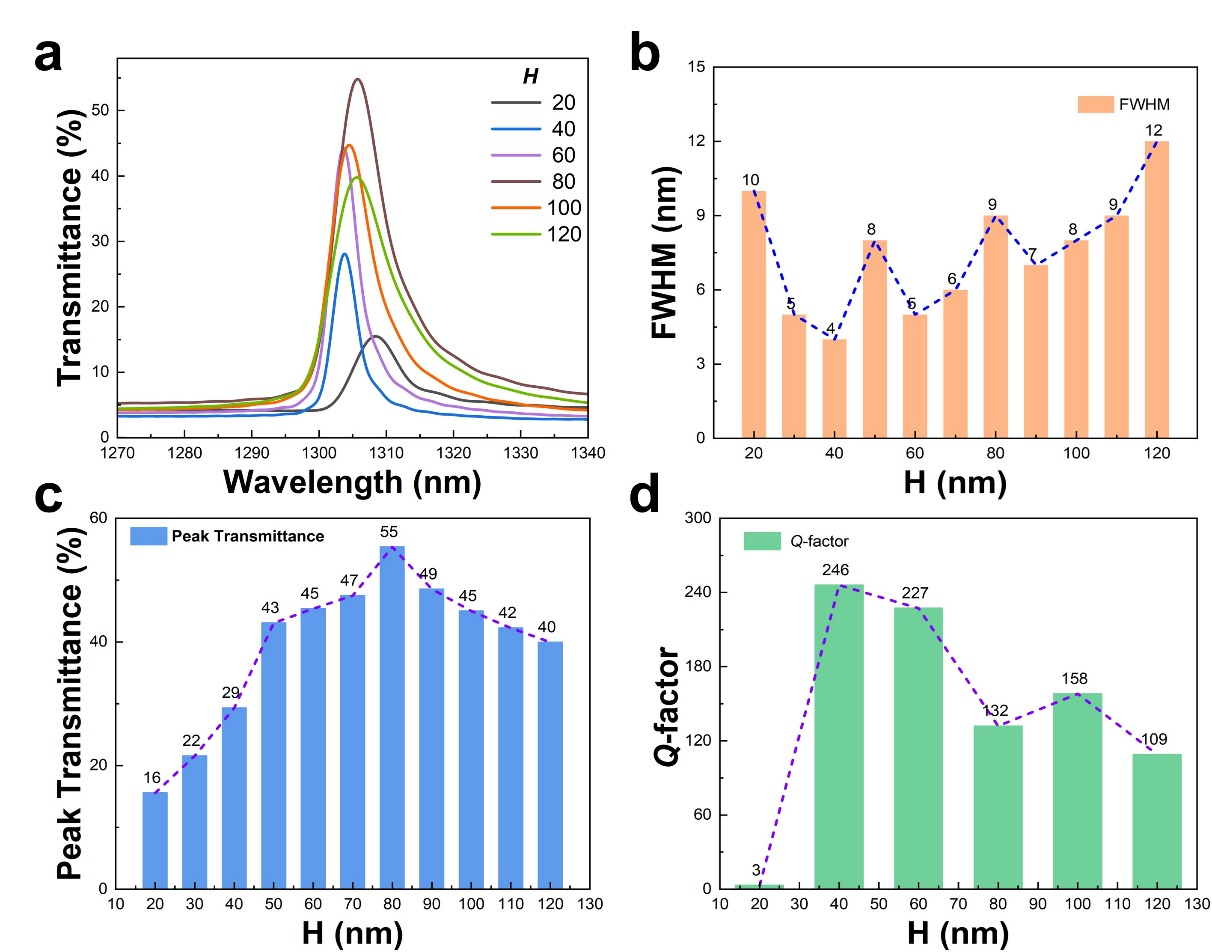


**Fig. S1 Depth-dependent resonance characteristics.** (a) Calculated transmission spectrum versus the nanohole cavity with depth variation *H* (20–120 nm). Fixed parameters: period *P* = 1500 nm, fill factor *FF* = 0.7, Au film thickness *t_Au_*= 25 nm. (b) Statistical full-width-at-half-maximum (FWHM) of transmission spectrum versus nanohole depth *H*. (c) Statistical peak transmittance of transmission spectrum versus nanohole depth *H*. (d) Statistical *Q*-factor of transmission spectrum versus nanohole depth *H*.

**S2. Metal film thickness (*t_Au_*) dependence analysis**

The thickness of the Au layer strongly impacts the resonance linewidth and quality factor through its effect on **surface plasmon polariton (SPP) excitation efficiency** and **Ohmic dissipation**. At very thin films (*t_Au_* <20 nm), the continuity of the metal is insufficient to support well-confined SPPs, resulting in weak coupling at the Au/ SiN_x_ interface and enhanced radiation leakage. This leads to broad transmission linewidths and reduced peak transmittance.

As the thickness increases, confinement of the plasmonic mode improves, reducing out-of-plane radiation loss and yielding narrower linewidths. An optimal balance is reached near *t_Au_* ≈30–35 nm, where coupling to SPPs is efficient while Ohmic absorption remains moderate. In this regime, the FWHM is minimized (~4 nm), and the transmission efficiency remains high. Beyond *t_Au_* ≳40 nm, increased metallic volume leads to stronger Ohmic dissipation, and the effective overlap between the incident field and SPP mode decreases. As a result, both the transmittance drops and the linewidth broadens (Fig. S2b). The *Q*-factor trend (Fig. S2d) reflects this interplay. With thickness increasing from 10 to 40 nm, the *Q*-factor rises steeply, reaching a peak value of ~278 at *t_Au_* =40 nm. Further thickening reduces the *Q-*factor slightly (to ~269 at 50 nm), due to excess Ohmic loss outweighing the benefits of improved confinement.

Overall, these results identify a practical **optimal thickness window of 20–35 nm**, which ensures robust SPP excitation, minimal linewidth, and high transmission efficiency. This range thus provides design guidance for high-performance, SPP-mediated plasmonic nanohole filters.


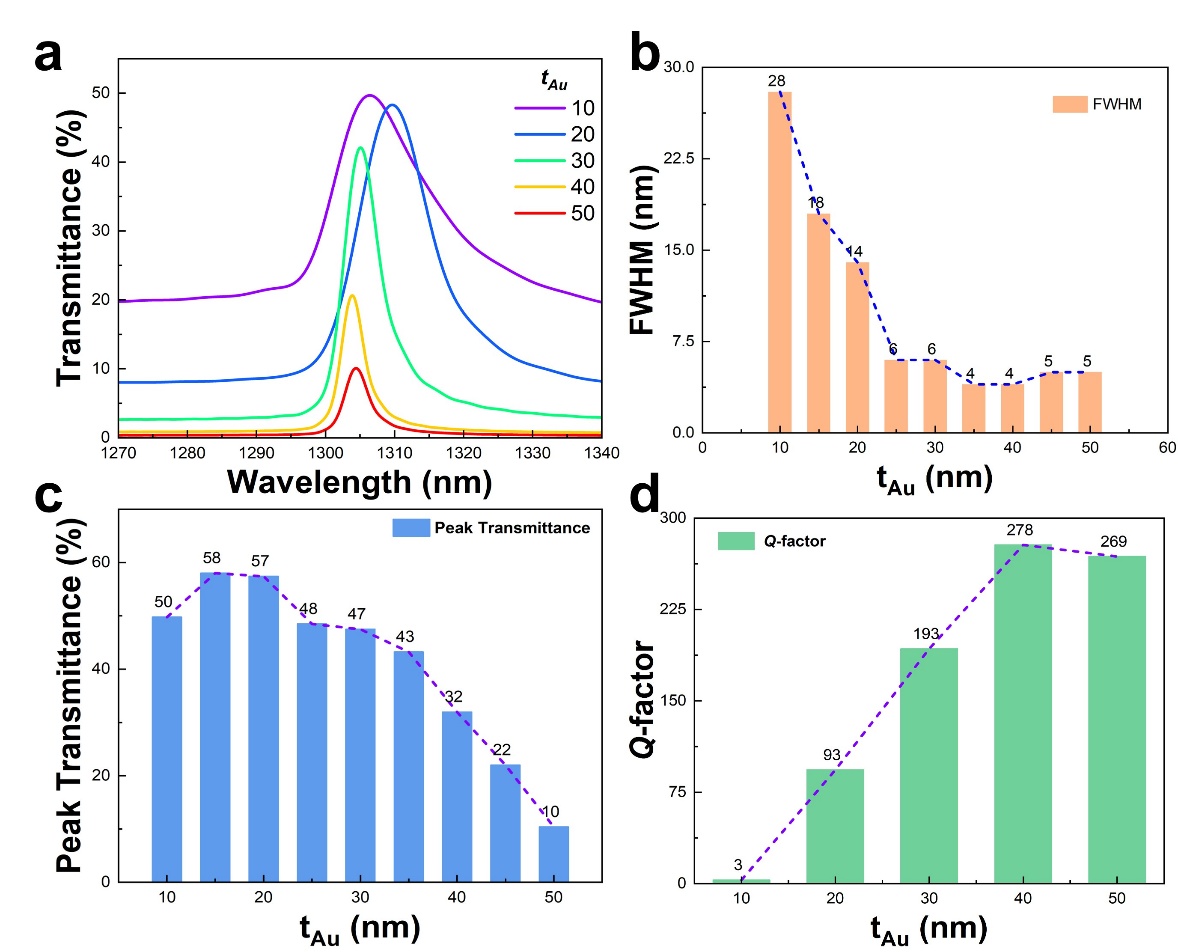


**Fig. S2 Au film thickness dependent resonance characteristics.** (a) Calculated transmission spectrum versus Au film thickness *t_Au_* (10–50 nm). Fixed: *P* = 1500 nm, *H*= 70 nm, *FF* = 0.7; (b) Statistical FWHM of transmission spectrum versus Au film thickness *t_Au_*. (c) Statistical peak transmittance of transmission spectrum versus Au film thickness *t_Au_*. (d) Statistical Q-factor of transmission spectrum versus Au film thickness *t_Au_*.

**S3. Fill factor (*FF*) optimization Analysis**

The fill factor *FF=d/P* (hole diameter-to-period ratio) governs the in-plane lattice coupling and the scattering strength of each aperture, thereby controlling how efficiently the periodic array supplies grating momentum to excite surface plasmon polaritons (SPPs) at the continuous Au/SiNx boundary. Unlike *t_Au_*, which primarily balances Ohmic dissipation against radiation leakage, *FF* tunes the lattice-mediated SPP coupling and the collective coherence of the array, which are key to resonance sharpening and high transmission. Fig. S3 reveals three representative regimes: First, under-filled lattice (*FF* = 0.3); Small apertures (large metal area) yield weak single-hole scattering and reduced lattice coupling, so grating-assisted SPP excitation is inefficient. The resonance therefore exhibits broader FWHM (>10 nm) and suppressed peak transmittance (~ 10%), with radiation leakage dominating due to poor overlap with the SPP-mediated mode. Second, balanced coupling (*FF* ≈ 0.7);The aperture scattering cross-section and lattice momentum are co-optimized: SPPs are efficiently excited and re-radiated through the holes, and boundary field enhancement is maximized while Ohmic loss remains moderate. This regime delivers narrow linewidths (FWHM ~ 5 nm) together with high peak transmittance (> 47%), reflecting strong collective coherence of the array near the grating-coupled resonance condition. Third, over-filled lattice (*FF* = 0.8); Very large apertures increase the metal–dielectric interface area participating in the mode and can introduce mode competition/spectral splitting between closely spaced lattice-supported resonances. Although the FWHM can remain small (~ 4 nm), the peak transmittance decreases (~38%), indicating that excess modal overlap and increased dissipative pathways offset the benefit of tighter confinement.

The *Q*-factor trend in Fig. S3d mirrors this interplay. As *FF* increases from 0.3 → 0.8, rises sharply and can reach ~ 370 at *FF* = 0.8 owing to linewidth reduction. However, the concurrent drop in peak transmittance signals diminishing returns for practical filter performance when *FF* is too high. Consequently, FF ≈ 0.7 emerges as a practical optimum, providing a favorable balance of narrow linewidth (high *Q-*factor) and high throughput. Design co-optimization with *t_Au_*. The *FF*-mediated lattice coherence synergizes with the *t_Au_* -mediated loss control: in the 20–35 nm thickness window, *FF* ≈ 0.7 compensates residual radiation leakage and sustains efficient SPP excitation; for thicker Au (> 40 nm), a slightly smaller *FF* can help mitigate growing Ohmic loss by reducing unnecessary modal overlap at the boundary. Treat *FF* not merely as a geometric ratio but as a lattice-coupling knob that regulates SPP excitation efficiency and modal purity. Together with *t_Au_*, it enables deterministic optimization toward the SPP-mediated, high *Q-*factor, high-throughput operating point summarized in Fig. 2(b).


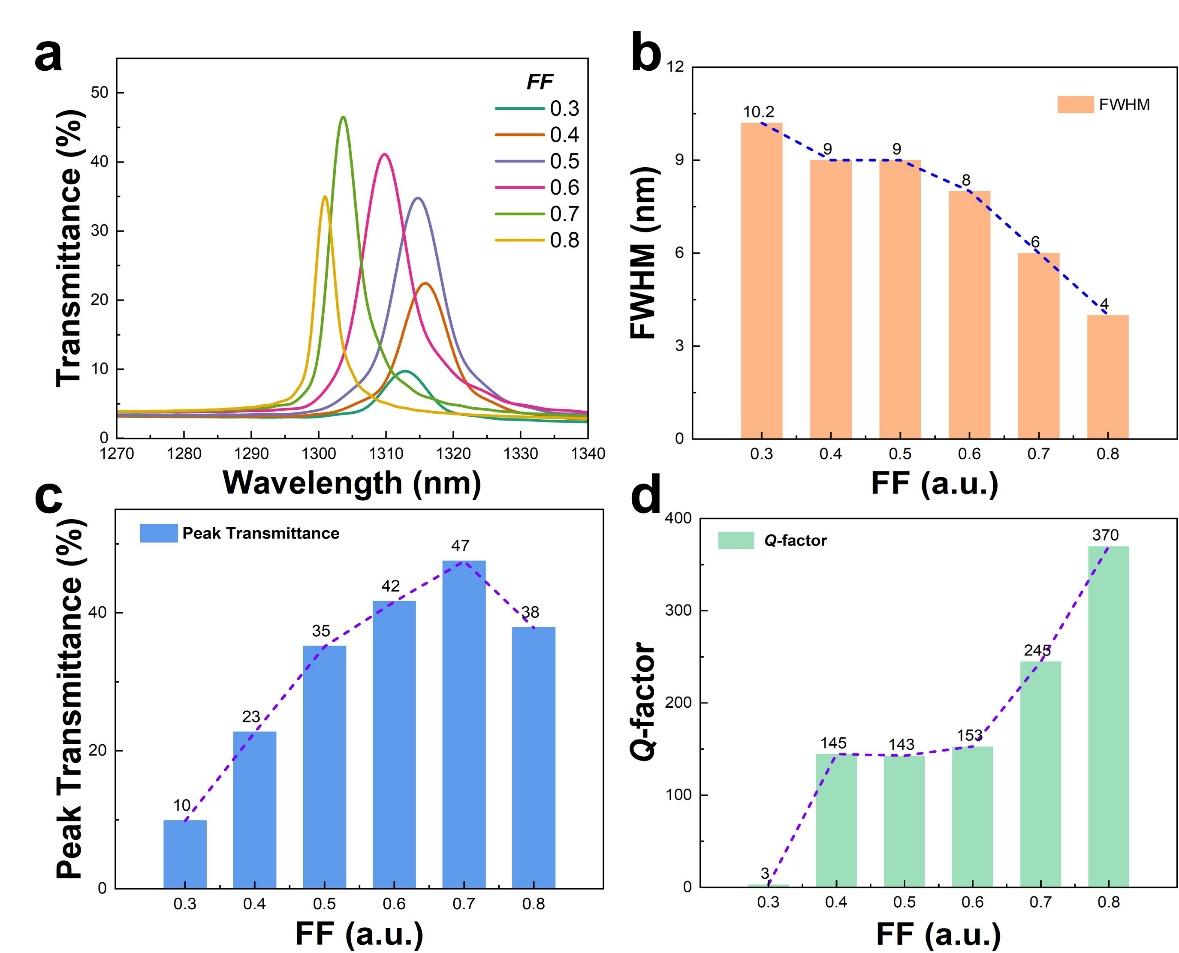


**Fig. S3 Fill Factor (*FF*) dependent resonance characteristics.** (a) Calculated transmission spectrum versus fill factor *FF* (0.3–0.8). Fixed: *P* = 1500 nm, *H*= 70 nm, *t_Au_* = 25 nm.; (b) Statistical FWHM of transmission spectrum versus fill factor *FF*. (c) Statistical peak transmittance of transmission spectrum versus fill factor *FF*. (d) Statistical *Q*-factor of transmission spectrum versus fill factor *FF*.

**S4. Near-infrared plasma transmission filter performance comparison table**

| Reference | Bandwidth (nm) | Tunable | | Min. FWHM (nm) |
| --- | --- | --- | --- | --- |
| This Work | 800 (900–1700) | period nanohole | 5 | |
| ^1^ | 270 (1510–1780) | period grating | 7 | |
| ^2^ | 800 (1100-1900) | periodic metal-dielectric-metal stack | ~200 | |
| ^3^ | 200(1400-1600) | liquid crystal metasurface (grating) | / | |
| ^4^ | 600 (1100-1700) | period grating | 7 | |
| ^5^ | 50 (1500-1550) | MIM heterostructure nanostrip arrays | ~10 | |
| ^6^ | 1500 (/) | Gate-Tunable  Metasurfaces | ~80 | |

**References**

1. E. Li, X. Chong, F. Ren and A. X. Wang, *Optics Letters*, **2016**, 41,1913.

2. Y. Liang, S. Zhang, X. Cao, Y. Lu and T. Xu, *Scientific Reports*, **2017**, 7,4357.

3. Y. Ni, C. Chen, S. Wen, X. Xue, L. Sun and Y. Yang, *eLight*, **2022**, 2,23.

4. Q. Zheng, X. Nan, B. Chen, H. Wang, H. Nie, M. Gao, Z. Liu, L. Wen, D. R. Cumming and Q. Chen, *Laser & Photonics Reviews*, **2023**, 17, 2300475.

5. J. Zhang, J. Yang, M. Schell, A. Anopchenko, L. Tao, Z. Yu and H. W. H. Lee, *Optics Letters*, **2019**, 44, 3653-3656.

6. Y. Lee, J. Yun, S. J. Kim, M. Seo, S. In, H. D. Jeong, S. Y. Lee, N. Park, T. D. Chung and B. Lee, *Advanced Optical Materials*, **2020**, 8, 2001256.
